# Landsat Surface Product Validation Instrumentation: The BigMAC Exercise

**DOI:** 10.3390/s25082586

**Published:** 2025-04-19

**Authors:** Dennis Helder, Mahesh Shrestha, Joshua Mann, Emily Maddox, Jeffery Irwin, Larry Leigh, Aaron Gerace, Rehman Eon, Lucy Falcon, David Conran, Nina Raqueno, Timothy Bauch, Christopher Durell, Brandon Russell

**Affiliations:** 1 KBR, Contractor to U.S. Geological Survey Earth Resources Observation and Science Center, Sioux Falls, SD 57030, USAjmann@contractor.usgs.gov (J.M.);; 2 U.S. Geological Survey Earth Resources Observation and Science Center, Sioux Falls, SD 57030, USA; jrirwin@usgs.gov; 3 Image Processing Lab, Engineering Office of Research, South Dakota State University, Brookings, SD 57007, USA; larry.leigh@sdstate.edu; 4 Chester F. Carlson Center for Imaging Science, Digital Imaging and Remote Sensing Laboratory, Rochester Institute of Technology, 54 Lomb Memorial Drive, Rochester, NY 14623, USA; adgpci@cis.rit.edu (A.G.); eoncis@rit.edu (R.E.); laf3991@rit.edu (L.F.); dnc7309@rit.edu (D.C.); ngrpci@cis.rit.edu (N.R.); tdbpci@rit.edu (T.B.); 5 Labsphere, Inc., North Sutton, NH 03260, USA; cdurell@labsphere.com (C.D.); branjrussell@gmail.com (B.R.)

**Keywords:** Landsat, surface reflectance/temperature, product validation, spectrometer, unmanned aircraft system (UAS), specular array radiometric calibration (SPARC), arable, TidbiTs

## Abstract

Users of remotely sensed Earth optical imagery are increasingly demanding a surface reflectance or surface temperature product instead of the top-of-atmosphere products that have been produced historically. Validating the accuracy of surface products remains a difficult task since it involves assessment across a range of atmospheric profiles, as well as many different land surface types. Thus, the standard approaches from the satellite calibration community do not apply, and new technologies need to be developed. The Big Multi-Agency Campaign (BigMAC) was developed to assess current technologies that might be used for the validation of surface products derived from satellite imagery, with emphasis on Landsat. Conducted in August 2021, in Brookings, SD, USA, a variety of measurement technologies were fielded and assessed for accuracy, precision, and deployability. Each technology exhibited its strengths and weaknesses. Handheld spectroradiometers are capable of surface reflectance measurements with accuracies within the 0.01–0.02 absolute reflectance units, but these are expensive to deploy. Unmanned Aircraft System (UAS)-based radiometers have the potential of making measurements with similar accuracy, but these are also difficult to deploy. Mirror-based empirical line methods showed improved accuracy potential, but their deployment also remains an issue. However, there are inexpensive radiometers designed for long-term autonomous use that exhibited good accuracy and precision, in addition to being easy to deploy. Thermal measurement technologies showed an accuracy potential in the 1–2 K range, and some easily deployable instruments are available. The results from the BigMAC indicate that there are technologies available today for conducting operational surface reflectance/temperature measurements, with strong potential for improvements in the future.

## 1. Big MAC Overview

### 1.1. Purpose

The USGS EROS Center widely distributes Landsat Surface Reflectance and Surface Temperature products to the remote sensing community. These relatively new products are produced by applying an atmospheric correction algorithm (LaSRC is used for Landsat 8 and 9) to standard top-of-atmosphere products to propagate pixel reflectance values to the Earth’s surface [1]. Although the calibration and validation of top-of-atmosphere, also referred to as Level 1, products is a highly developed process that has been ongoing for several decades [2,3,4,5,6,7], validation of land surface products is still in its early stages.

Several aspects of land surface product validation combine to make the task substantially different from the calibration of satellites [8]. First, validation must include multiple surface cover types, and the multitude of spectral signatures of surface targets requires validation at multiple locations across the planet [9,10]. Secondly, the atmospheric correction process also needs to be validated across a variety of atmospheric optical parameters. Thirdly, instrumentation is needed to make the appropriate surface and atmospheric measurements to perform proper validation. Thus, there is a great need to optimize the validation process to ensure the maximum return of information is gained for a fixed level of investment.

Surface product validation is not limited to only Landsat sensors, and many organizations worldwide are directing resources to improve surface product validation [9,11]. Geoscience Australia has been leading the effort through field campaigns using portable spectrometers and sun photometers to validate products on a continental scale [12]. In Europe, the approach has developed around the concept of in situ automated hyperspectral radiometers [9,10,13]. Multiple approaches can be used to validate surface products; however, an optimal approach has not yet been determined.

**Figure 1:**
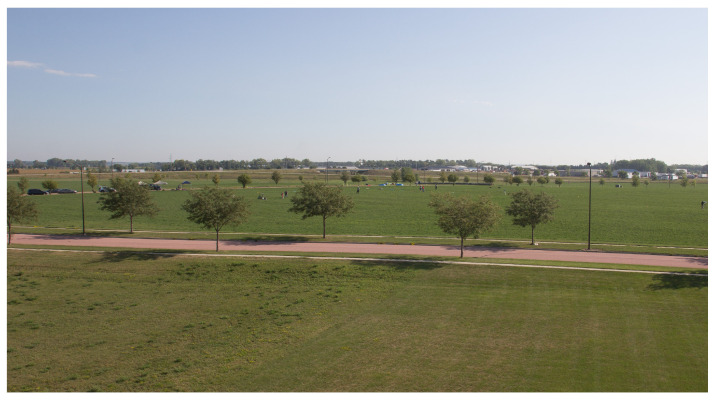
View of the BigMAC site.

**Figure 2:**
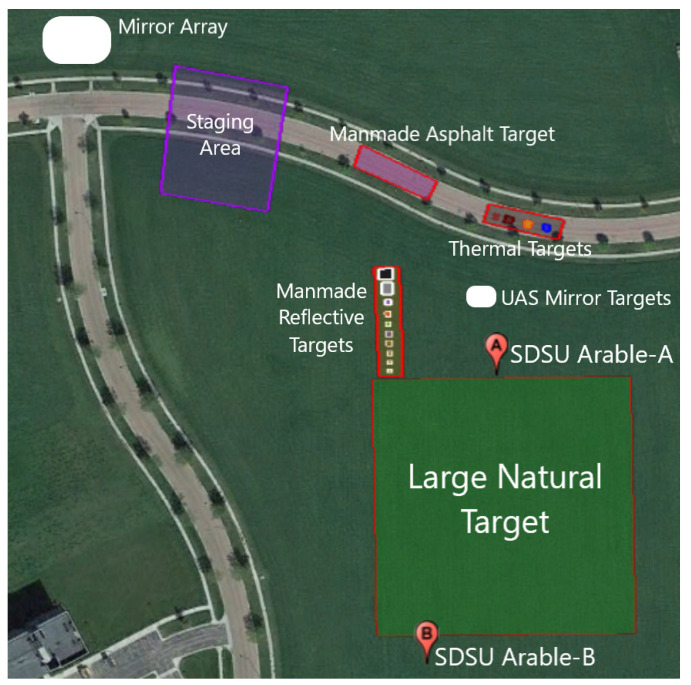
Location of the various targets used in the BigMAC activity.

To address this problem, USGS EROS conducted a multi-agency field campaign (titled BigMAC) during 30 August–3 September 2021 in Brookings, SD, USA, at the Research Park for SDSU. This 125-acre facility has large uniform regions of alfalfa that provide a natural vegetative background target along with asphalt roads for a common human-made target. In addition, a number of small panels were deployed with a variety of reflectance and spectral signatures, as well as several thermal targets. Figure 1 shows the site, while Figure 2 shows a pictorial layout of the various targets.

Four organizations participated in BigMAC: USGS EROS, SDSU, RIT, and Labsphere. Each organization provided targets, instrumentation, and personnel for the campaign.

#### 1.1.1. Targets

**Figure 3:**
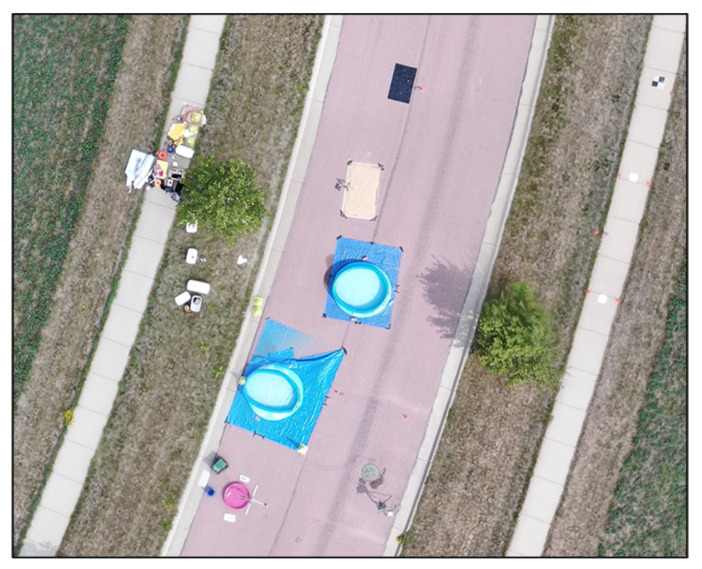
Color image of thermal water and sand targets.

**Figure 4:**
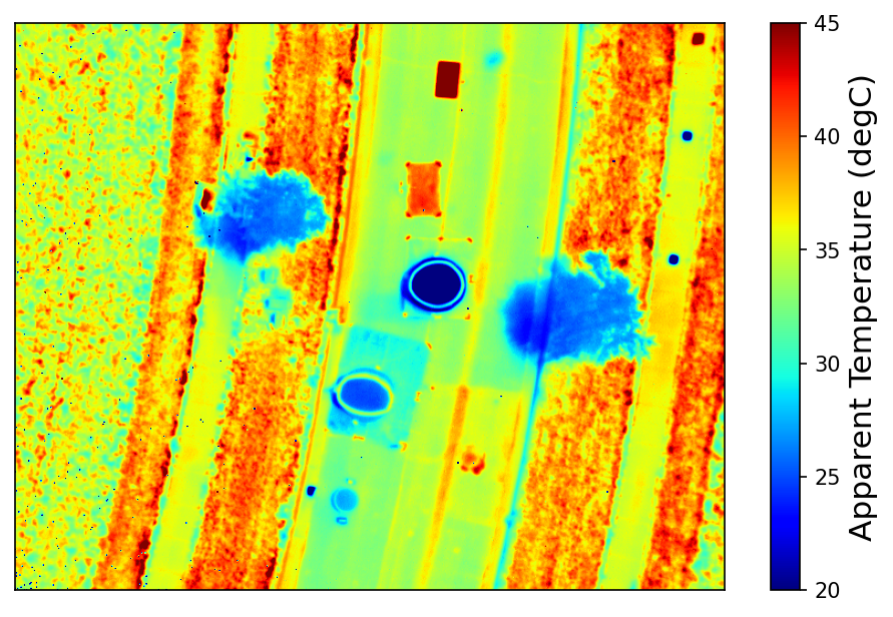
Colormapped thermal image of water and sand targets.

A variety of targets were developed for the campaign. Since the campaign focused on surface reflectance and temperature measurements, most targets could be developed at a small scale for fabrication and deployment. Figure 2 illustrates an array of human-made reflective targets. These included felt radiometry panels and spectral panels developed by RIT, two Permaflect panels developed by Labsphere, and two ‘mystery panels’. Unlike the rest of the human-made reflective panels, the ‘mystery panels’ were obtained from an organization not participating in the BigMAC—University of Arizona Remote Sensing Group. Thus, they served as blind targets to the participants to eliminate bias as much as possible for at least a small subset of the targets that were used. A large, 100 m by 100 m, portion of the alfalfa field served as a large natural target, as indicated in Figure 2. The small human-made reflective targets provided an opportunity for measuring precision and repeatability in a more controlled environment, while the large natural alfalfa target represented what would be a more ‘normal’ measurement in the operational assessment of Landsat surface product validation. Alfalfa is advantageous in that it represents agricultural targets and is short enough so that reflectance can be measured using handheld or backpack-based spectrometers. This particular field had just been mowed and baled for hay the previous week. So, the field was optimally short and greening up well after the mowing. A disadvantage of using alfalfa as a convenient natural target is that the plant canopy is substantially different than forests and other taller vegetation. Additional targets included a portion of an adjacent asphalt roadway which served as a very stable larger target to assess repeatability, and a set of thermal targets–warm and cool pools of water, sand, and rubber mats. The water pools were used because the emissivity of water is well understood, and water tends to be fairly stable with temperature over time. The sand and rubber mats represent a more natural target and a reasonably stable non-water target. Figure 3 and Figure 4 show the thermal targets in both the reflective and temperature space, respectively. Thermal measurements were taken for the large natural alfalfa target as well.

#### 1.1.2. Measurement Technologies

The immediate purpose of BigMAC was to assess multiple measurement technologies with respect to accuracy, precision, and deployability. Both the instruments and platforms for the instruments were assessed.

Perhaps the most often used instrument to measure surface reflectance in the community involved with calibration of optical remote sensing satellites is the handheld spectroradiometer. Three teams brought the following instruments to BigMAC: USGS EROS, SDSU, and RIT. Each of them used models of the ASD FieldSpec instrument, commonly referred to as an ASD. These instruments have been used for decades and are quite well understood with regard to accuracy and repeatability [14,15]. In general, two person teams are required and the initial cost of the instrument is significant. Typically, these instruments are calibrated through observing a well-characterized reflectance panel and are then used to measure unknown surface reflectances in the visible through SWIR wavelengths. The dual spectrometer approach uses two instruments operating in tandem at the field site. One spectrometer continuously monitors the reflectance panel while the other measures the unknown target [16]. One of the difficulties when using a reflectance panel as the calibration source for the ASD is that the reflectance of the panel is transferred to the radiance measurement of the ASD. Thus, while the ASD is being used to measure the target, any changes in downwelling irradiance will cause a change in the radiance recorded by the ASD that is indicative of an illumination change and not a surface reflectance change. Simultaneous observation of the reflectance panel with a stationary ASD can be used to track illumination changes during site measurements, and these changes can be transferred to the mobile unit during post-processing. In this manner, changes in atmospheric transmission can be tracked continuously to reduce variability during the measurement of the unknown target. USGS EROS deployed a dual unit system [11,17], while SDSU and RIT fielded single unit systems. However, the RIT instrument was primarily used to observe the full hemispherical downwelling irradiance with a RCR to track atmospheric changes for their UAS instrumentation and Labsphere is a mirror-based demonstration.

During each collection activity, the handheld radiometer teams would measure the asphalt target, followed by the row of human-made reflective targets and then collect data over the large natural alfalfa target. Collection patterns over the large target were left up to individual teams—SDSU collected north/south transects following their standard operation procedure, while USGS EROS collected along east/west transects which crossed the rows of alfalfa rather than paralleling them.

In 2017, RIT established an Unmanned Aerial Systems Laboratory [18] along with a field team capable of deploying UAS-based imaging systems throughout North America. This UAS team conducts hundreds of missions annually, for studies ranging from harmful algal blooms, the detection of vegetative diseases, target detection, and the calibration of sensors.

**Figure 5:**
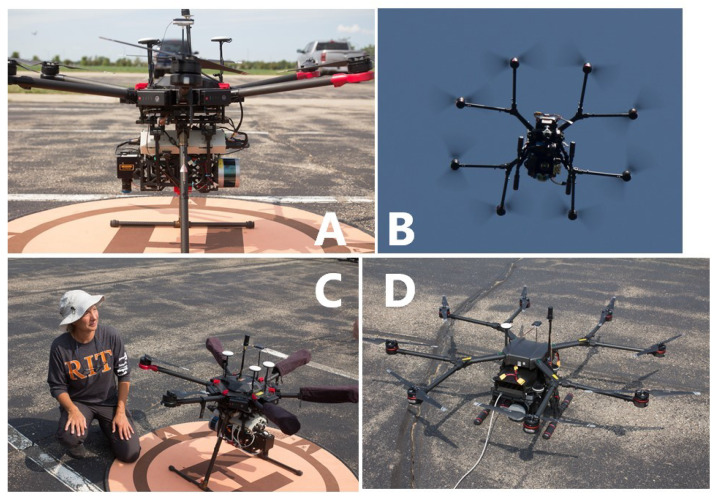
Photos of the MX-1 (**A**,**C**) and MX-2 (**B**,**D**) UAS.

As part of the BigMAC, RIT flew four unique UAS as follows: the MX-1, SWIR, MX-2, and DJI Mavic 2 Enterprise. The MX-1 is a multi-modal sensor payload that simultaneously captures Nano hyperspectral (400–1000 nm), uncooled thermal, three-band color, and five-band multispectral imagery, as well as LiDAR and GPS/IMU data; see Figure 5A,C [19,20]. The SWIR payload was also flown in order to extend the spectral range out to 2500 nm. The MX-1 and SWIR sensor payloads were flown on two separate DJI Matrice 600 Pro UAS platforms in tandem. The MX-2 payload consists of the same capabilities as MX-1 with the following two critical upgrades: a calibrated cooled thermal FLIR A6751sc SLS imaging system and an UAS platform with more lifting power, the DJI Wind 8; see Figure 5B,D. Finally, a DJI Mavic, with a high spatial resolution RGB camera was flown in order to produce an overall basemap of the entire site.

Pertinent to the reflective portion of this campaign were two Headwall sensors—the Headwall Nano and Headwall SWIR M384 sensor—which, together, cover from 400 to 2500 nm in 2.2 nm and 10 nm increments, respectively [19]. The two UAS platforms flew parallel flight lines at 200 ft AGL, capturing the entire site shown in Figure 2. Image and GPS/IMU data were processed and georectified at RIT, the WGS84 coordinate system was used as the geodetic reference, and AeroPoint ground control points (GCPs) were employed for geometric correction to ensure the accurate georeferencing and spatial alignment of the imagery. Then, the UAS images were sent to SDSU for radiometric calibration, target extraction, and surface reflectance estimation for all targets. Radiometric calibration of the imagery was performed using the SDSU-measured reflectance of the Labsphere Permaflect panels to perform an empirical line-based calibration [21].

Pertinent to the thermal portion of this campaign, the MX-2 carries a cooled FLIR A6751sc SLS thermal imaging sensor. The FLIR is a broadband radiometric temperature imaging sensor with a spectral range of 7.5–10 microns and was flown over the entire BigMAC site [20].

In addition to the UAS sensors, RIT also fielded several ground-based instruments for measuring surface temperature. Chief among these was a FTIR spectroradiometer, called the μFTIR (developed by Designs & Prototypes). The μFTIR is designed to collect spectral measurements from 2 to 16 µm at a resolution of 6 cm^−1^, and it has a measured noise equivalent delta temperature (NEΔT) of less than 0.3 °C. The Hobo MX2204 TidbiT water temperature logger was used in the thermal pool targets [22]. These small units have an advertised accuracy of ±0.2 °C over the operational range of 0–70 °C.

The SPARC method employs convex mirrors to relay the image of the full solar disk to a sensor under test. Originally SPARC was developed as geometric and spatial reference points for deriving the point spread functions and modulation transfer functions of the sensor under test. However, SPARC also produces targets suitable for deriving the absolute calibration coefficients of remote sensing Earth systems in the solar reflective spectrum [23,24]. By varying the number of mirrors in a target, as well as the reflectance and RoC, targets can be produced for a wide variety of at-aperture radiance levels suitable for airborne- or satellite-based sensors of different resolutions from centimeters (e.g., UAS) to larger footprints in the hundreds of meters or above. It should be noted that because the SPARC mirrors are designed to deliver reflected light from the full solar disc to the sensor aperture, the diameter of the SPARC mirror has no impact on the at-sensor radiance level produced by the target. The diameter only impacts the targets projected angular FOR at which the full disk of the sun is visible by the overflying sensor. Based on the sky conditions, the SPARC algorithm can also correct for any significant hemispherical sky radiance reflected by the convex mirror based on radiative transfer modeling or the ground measurements of the diffuse-to-global ratio (G) at the time the target is imaged and the known FOR [23].

As part of the BigMAC experiment, a number of mirror targets were deployed for the calibration and characterization of UAS and other satellite assets alongside the traditional diffuse reflectance techniques [25]. With the precise knowledge of mirror properties, atmospheric modeling, and appropriate measurements, it is possible to derive the absolute radiance associated with a SPARC target and a given sensor. The mirrors then serve as a SI-traceable point source [26]. A key factor of the SPARC design is that the full solar disk virtual image from each mirror is smaller than the mirror diameter and much smaller than the sensor IFOV, thus producing nearly ideal point sources but free of significant diffraction effects that would plague other methods utilizing pinhole point sources for solar calibration. Similarly, and of great utility for correcting imagery to bottom-of-atmosphere surface reflectance [25,27], it is possible to derive a LER for a SPARC target. The LER represents the bidirectional reflectance factor corresponding to the ratio of the radiance reflected by the SPARC target in the particular direction of the imaging sensor to the radiance that would be reflected into the same direction by an ideal Lambertian target, with identical illumination having the area equivalent to a single-pixel. The ratios are defined so that an ideal Lambertian reflector has a total reflectance factor of 100%. It is important to note that a true Lambertian material cannot have a reflectance greater than 100%. However, because the SPARC target is specular, it can reflect more light in a given direction than an ideal Lambertian reflector. Thus, it is possible for a SPARC target to produce a LER much greater than 100% by selecting a sufficient mirror RoC. The reader will notice that this will be true for the SPARC reflectors used in this study to maximize the measured signal-to-noise radiance response but still avoid saturation nonlinearity.

**Figure 6:**
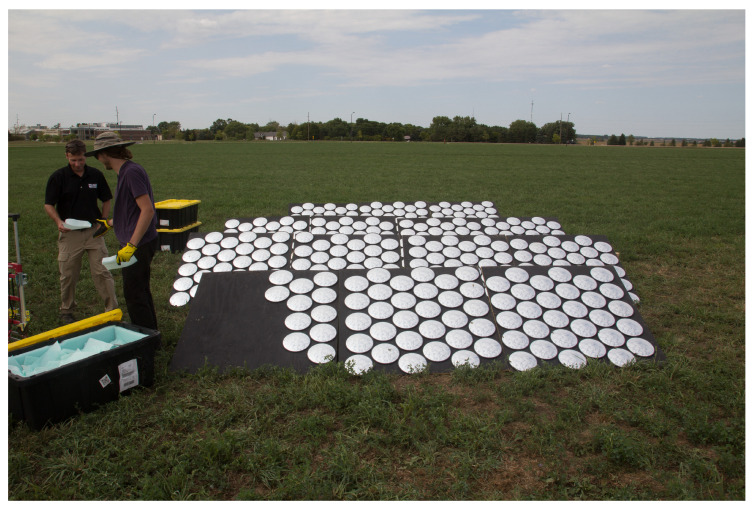
Mirror array used to replicate a bright point target.

Two separate iterations of SPARC were used during BigMAC. A number of manually placed target arrays made of multiple mirrors were placed at the primary experiment site; Figure 6. These “manual” arrays were individually pointed by hand based on a set observation geometry determined by the solar azimuth angle at the measurement time and the predicted view angle of the sensor under test. Secondly, an automated version of SPARC was utilized. The FLARE represents a commercial, on-demand calibration network [26]. A network node was located in Arlington, SD, 30.8 km WNW from the BigMAC experiment site. This node is designed for GSD up to 60 m, and it was tasked with providing calibration against Planetscope and the assets available during the experiment.

During field measurements, an ASD FieldSpec 4 spectrometer (Malvern Panalytical Ltd., Malvern, United Kingdom) with RCR foreoptic was deployed to measure downwelling irradiance over the course of the experiment. Measurements were made for the full sky global irradiance, with discrete shaded measurements separating the global, direct, and diffuse components. The direct solar irradiance was utilized to derive the atmospheric optical transmission and at-aperture radiance for the SPARC manual mirrors and automated FLARE node, while the diffuse-to-global irradiance ratio was utilized in calculating LER (λ) [23,27].

A common approach in the vicarious calibration of Earth Observing systems is the ELM [21,27,28]. Two or more targets of known radiance (or reflectance) are presented to a sensor under test and the sensor’s response is assessed. By performing regression against multiple radiance levels, a calibration gain and offset can be produced across the sensor’s linear response range. An important consideration in ELM is the RAIFoV of the system under test [29]. The calibration targets must be large enough to contain pixels that are not contaminated by edge or adjacency effects. During BigMAC, multiple reflectance levels of grayscale tarps were used to perform ELM for the high-resolution UAS systems. However, these targets were not large enough to provide any radiometrically accurate pixels for the coarser resolution satellite systems. Leveraging the scalable SPARC methodology, targets of multiple at-aperture radiance or LER levels were used for a MELM regression applicable to UAS and Sentinel-2A MSI imagery [30].

Arable (San Francisco, CA, USA) makes a low-cost field-deployable radiometer, known as the Arable Mark 2, primarily for agricultural applications. It has a multi-band downwelling and upwelling radiometer with set spectral filters covering the VNIR portion of the solar spectrum. This instrument is meant to be deployed throughout the agricultural growing season, is solar powered, and uploads data automatically via a cellular network interface. Details on the characterization and calibration of these units can be found in [31]. SDSU has used a number of these units over the past two years in an effort to evaluate their stability and accuracy for satellite calibration and validation [32]. Two units were deployed during BigMAC, as shown in Figure 2. The design of the large natural alfalfa target to border the Arable field of view allowed the efficient collection of data for ASD-based systems and Arables.

#### 1.1.3. Field Conditions

The large natural target, shown in Figure 2, was an alfalfa field that was harvested approximately one week before August 30 and 31, the field collection period. The alfalfa had grown back to approximately 4 inches (10.16 cm) in height.

**Figure 7:**
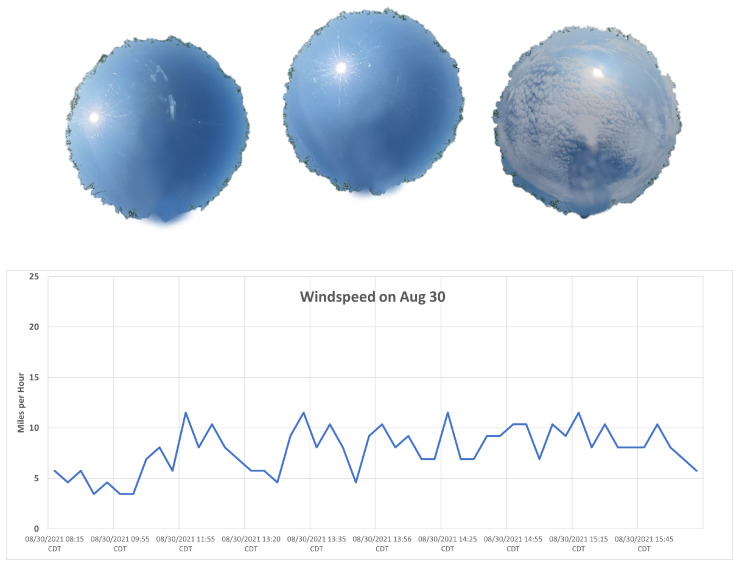
Weather conditions on 30 August 2021. All-sky photos are shown on the top row for approximately 10:30 AM (15:30 UTC), 12:30 PM (17:30 UTC), and 2:30 PM (19:30 UTC), respectively. A timeline of the wind speed recorded by the Brookings airport ASOS station is shown in the lower figure.

Weather conditions varied throughout 30 August 2021. Temperatures remained in the upper 70 °F throughout most of the day, reaching a maximum temperature of 80 °F. The 10:00 AM (15:00 UTC) and 12:00 PM (17:00 UTC) field collection periods were free from clouds (see Figure 7). Broken altocumulus clouds formed over the Brookings area in the early afternoon, providing varied cloud cover during the 2:00 PM (19:00 UTC) field collection period, as shown in the upper right image of Figure 7. Wind speed data were taken from the Brookings Airport ASOS. Winds remained calm throughout the 10:00 AM collection period, with winds around 5 mph, as shown in the lower plot of Figure 7. Winds began to increase at the 12:00 PM field collection period, fluctuating around 9 mph ± 2 mph.

**Figure 8:**
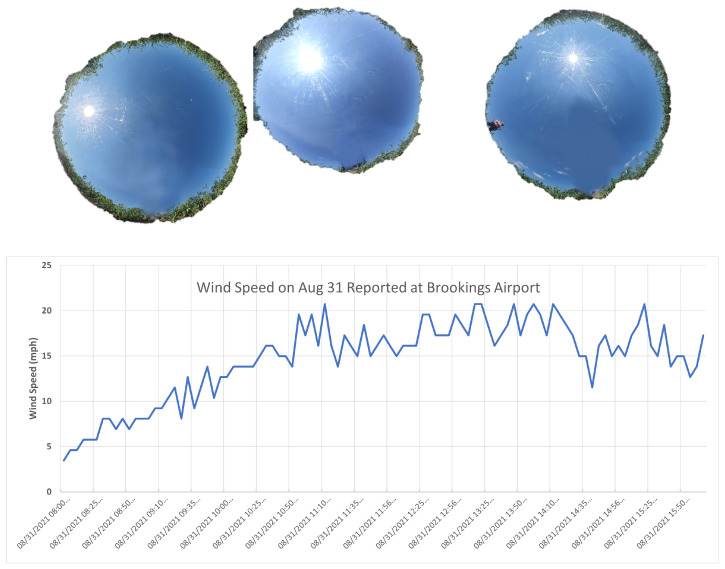
Weather conditions on 31 August 2021. All-sky photos are shown for approximately 10:30 AM (15:30 UTC), 12:30 PM (17:30 UTC), and 2:30 PM (19:30 UTC), respectively. A timeline of the wind speed recorded by the the Brookings, SD airport ASOS station is shown in the lower figure.

Temperatures on 31 August were once again around 70 °F, with a maximum temperature of 78 °F. Cloud cover on 31 August 2021, remained clear throughout the majority of the field collection periods, as shown in Figure 8. Small cumulus clouds began to develop during the final field collection period, as shown in the top right image of Figure 8. Winds were higher throughout the day, with winds of 12 mph at the start of the 10:00 AM field collection period, continuing to increase throughout the hour. The 12:00 PM field collection period winds varied from 15 mph to 20 mph. Therefore, it was decided that the UAS would not be used at the final 2:00 PM collection time. While there was a very brief moment of the winds dropping below 15 mph from 2:00 PM–3:00 PM, winds remained at 15 mph–20 mph throughout the rest of the hour.

## 2. Data Collected

Data were collected in the field at the Research Park for SDSU on Monday, 30 August 2021, and Tuesday, 31 August 2021. Weather conditions, as mentioned previously, ranged from clear skies to broken clouds, winds ranged from 5 to 20 mph, and temperatures were in the 70s and 80s Fahrenheit. All teams had the equipment set up by 9:30 AM and most work was finished by 3:30 PM.

### 2.1. Reflective

For surface reflectance measurements, efforts were focused on three specific times of day as follows: 10:30 AM, 12:30 PM, and 2:30 PM local time. These three times were chosen to represent typical morning and afternoon satellite data overpass times, and a time in between close to solar noon when change in solar illumination during data collection would be minimal was also chosen. All teams using ASDs, as well as UAS flights, would begin at these times so that measurements were made within a few minutes of each other. Thus, all measurements were made with substantially the same illumination conditions. All ASD measurements were made following the protocols described in [15,17] from a nadir-viewing geometry; that is, the center line of their field of view was directed normal to the Earth’s surface and the UAS followed the ASD measurement sequence. Table 1 provides details for all flights conducted during BigMAC.

Surface reflectance data were all recorded with hyperspectral sensors at essentially 1 nm resolution. However, since the primary emphasis of the BigMAC campaign was to determine the best procedures for measuring surface reflectance for the evaluation of Landsat products, all hyperspectral data were banded into Landsat 8 OLI reflective bands. These banded surface reflectance values were calculated on both days, all three acquisition times, for all reflectance targets, and for each OLI band (except the Cirrus band which normally is unable to view land surfaces). The mean values and standard deviations of the data were recorded and compared for each measurement methodology. Overall means and standard deviations across all days and all teams were also recorded. Key measurements of interest were consistency in measurements performed in the mornings, at noon, and in the afternoon to determine what might be expected for the operational precision of the surface reflectance measurements. In addition, certain targets—the Permaflect panels and the ‘Mystery’ panels—were absolutely calibrated by independent laboratories, and the measurements of these panels were evaluated for absolute accuracy.

### 2.2. Thermal

Thermal data collected via the MX-2 UAS essentially followed the same acquisition protocol as the reflective measurements. However, in addition to acquiring thermal imagery of the entire test site via parallel flight lines during morning, noon, and afternoon acquisitions, there were also opportunities for the UAS to hover over thermal targets of interest and ‘stare’ at the targets for an extended time. The temperature of land, vegetation, and some human-made objects can change substantially with a slight change in wind speed or solar loading. Water, however, is an ideal thermal target, as it is temporally stable and has a known emissivity. These ‘stare’ tests focused on the deployed thermal targets, specifically the warm and cold water baths, to assess the calibration and stability of the in-flight performance of the FLIR.

Ground-based temperature sensors followed a less prescribed measurement schedule. The TidbiT water temperature sensors recorded data continuously and automatically, every 30 s, throughout the entire day in the water pools. The μFTIR requires several minutes of integration time to acquire spectral measurements. Thus, it also recorded temperature information almost continuously throughout each day as it was moved from target to target. However, the μFTIR deployment was coordinated with the UAS FLIR measurements to obtain simultaneous measurements over specific targets. The measurement protocol for the μFTIR is further detailed in [33].

## 3. ASD and UAS Technology Assessment

Two key assessments for this project were the precision (how close I can come to obtaining the same measurement each time) and the accuracy (how close my measurement is to the true value) of the measurement technologies. Because of the fundamental difference in the measurement of surface reflectance versus surface temperature, temperature and reflectance will be addressed in separate sections.

### 3.1. Precision

A key attribute of land surface reflectance and temperature measurements is precision or repeatability. It is imperative to know that the same measurement is being made across instruments, operators, and time. The degree to which ASD- and UAS-based measurements are repeatable is the focus on this section.

#### 3.1.1. Data Reduction

Because of the large amount of data that was collected (15 targets × 8 instruments × 7 spectral bands), it is necessary for this presentation to slightly reduce the scope. Therefore, only results from three Landsat 8 spectral bands will be presented as follows: Green (525–600 nm), NIR (845–885 nm), and SWIR2 (2100–2300 nm). Results from these three bands are nearly identical and representative of all OLI bands. Furthermore, only a subset of targets will be used in the analysis. For each band, a set of 3–4 brightest and a set of 3–4 darkest targets were selected. These targets represent the end points in the reflectance range often observed by satellite sensors, and differences in repeatability can be scaled between these extremes.

Repeatability is important at several different levels. First, given the same instrument and operator, how repeatable is the measurement? Secondly, given that one instrument/operator cannot cover all locations, is there a decrease in repeatability when multiple instruments/operators are used? Third, how do UAS-based measurements compare to ASD-based measurements? Do they exhibit the same degree of precision?

All units in the following tables are in absolute reflectance units. While percentages are often easier to evaluate and understand, units based on percentages can be misleading for low reflectance values where percentage-based differences can become quite large. Secondly, for the purposes of the evaluation of land surface products, the aim is to know the absolute deviations from the true values in order to properly evaluate the accuracy of the product.

#### 3.1.2. Green Band

Table 2 shows the results for the Green band for each ASD system individually. In this case, there are three systems. USGS EROS used a dual ASD system. This system employs one ASD to stare at a reflectance calibration panel while the other ASD is a mobile unit with an operator collecting data. However, USGS EROS processed the data collected by the mobile unit as if it were a single unit system, as well as a dual unit system. This is noted as ‘EROS-Single’ and ‘EROS-Dual’ in the table. SDSU used a traditional single unit system.

**Figure 9:**
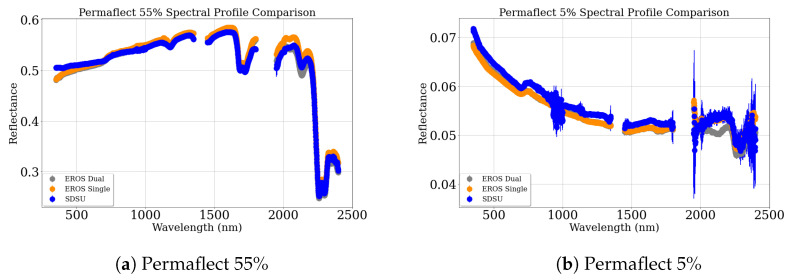
Spectral profile comparison of Permaflect targets.

Four bright targets were used for this band as follows: Permaflect 55%, RIT 30%, Asphalt, and Mystery Gray. In this case, bright targets ranged from 30% reflectance to 51%. Similarly dark targets were Permaflect 5%, RIT 2%, Alfalfa, and Mystery Black. These targets ranged from 2% to 6% reflectance. Figure 9 compares the full range spectral profile (with 1-sigma error bars) of Permaflect 55% and 5% targets, representing a bright and a dark target, respectively. The spectral profiles of both Permaflect 55% and 5%, measured using the EROS and SDSU spectrometers, are similar.

To determine the repeatability, the key data point was the difference recorded between morning (AM) measurements and noon measurements, as shown in Table 2. Afternoon measurements were not utilized due to the extreme sky cloud cover on the afternoon of the first day of the campaign. Ideally, since all instruments/operators were measuring the same target, at the same time, two days in a row, zero difference would be recorded. In this case, differences, on average, tended to be 0.015 ± 0.01 for bright targets and 0.002 ± 0.001 for dark targets. In addition, morning differences are slightly larger than noon differences. This is likely due to the greater movement of the sun earlier in the day combined with the BRDF of the targets. Of special interest is the alfalfa target since it represents large vegetative targets that are likely to be used in operational land surface reflectance measurements. Results here tend to be similar between the alfalfa target and the various artificial targets.

Table 3 shows the results for the Green band across all ASD systems. Again, only the morning (AM) and noon differences are used. In this case, the mean of all measurements across all systems is shown along with the standard deviation of those measurements. The standard deviation reflects the repeatability that is achievable across multiple teams. Repeatability is ±0.01, in general, for bright targets and ±0.001 for dark targets.

UAS repeatability in the Green band is shown in Table 4. The same target set is used, along with the morning and noon differences. Noon differences seem to be a bit smaller than the morning differences, likely due to target BRDF. The repeatability of bright target measurements varies from 0.004 to 0.058 ±0.02, while for dark targets it is 0.001 to 0.017 ± 0.005.

On average, UAS bright and dark target repeatability is somewhat larger than that for the ASD. However, the repeatbaility is comparable excluding Mystery Gray target.

#### 3.1.3. NIR Band

Table 5, Table 6 and Table 7 show the repeatability results for the NIR band. In the case of this spectral band, the bright targets were the same, except the RIT Red target replaced the asphalt target for a range of reflectance from 0.38 to 0.53. Only three really dark targets existed for this band—Permaflect 5%, RIT 2%, and Mystery Black. For the individual ASD system, bright (0.4 to 0.6 reflectance) target repeatability is 0.014–0.020 ±0.01 and darker (0.02 to 0.06 reflectance) target repeatability is 0.001–0.002 ±0.001. From Table 6, results across all ASD systems indicate repeatability for bright targets from ±0.01 to 0.02 and for darker targets ±0.001. The results for the UAS indicate that brighter target average reflectance values were quite consistent with the ASD values. However, bright target repeatability ranged from 0.01 to 0.04. In comparison to all ASD teams, bright target uncertainty (standard deviation) is almost double for the UAS but is very similar if the Mystery Gray target is removed. Dark target average reflectance measurements were quite different than ASD values, and dark target repeatability was also larger as follows: 0.005 versus 0.001. Interestingly, repeatability was the highest for the Permaflect target.

#### 3.1.4. SWIR2 Band

Table 8, Table 9 and Table 10 provide the results for the SWIR2 band. For this band, there is repeatability for individual systems and bright targets from ±0.01 to 0.02, while for dark targets the repeatability is from ±0.001 to 0.002. Across all teams, the repeatability is the same as for individuals. That is, it does not matter if only one instrument/operator combination or multiple instrument/operators are available because the repeatability is essentially the same. Unfortunately, the UAS surface reflectance measurements in the SWIR2 band experienced difficulties. The small human-made targets only provided a few pixels resulting in large uncertainties in those measurement extractions (indicated by the asterisks in Table 10). UAS hyperspectral data were acquired using two separate optical systems. Due to the SWIR system’s larger GSD, nearly twice that of the VNIR system, extracting SWIR channel data from UAS imagery was difficult and unreliable. Therefore, UAS SWIR channel data for the mystery panels were omitted from the analysis. Interestingly, the Permaflect targets were somewhat larger and reflectance estimates from them were quite reliable. In fact, they were quite similar to the ASD estimates.

#### 3.1.5. Summary

In summary, the particular wavelength of measurement really made no difference in repeatability for the ASD-based systems. In general ASD-based systems can measure brighter targets with a precision of 0.01–0.02 and darker targets at a precision of 0.001–0.002. UAS-based systems using the Headwall sensors perform very comparable to ASD-based systems for bright targets. However, for dark targets, UAS precision was closer 0.005 reflectance units. Large, pristine panels, such as the Labsphere Permaflect targets, exhibited better precision than the other small targets.

Due to the stable atmosphere during the morning and noon collection times on both days, little difference could be seen between the EROS single and double ASD approach.

### 3.2. Accuracy

The accuracy of the surface reflectance measurements is a primary factor for the operational validation of surface reflectance products. Four targets in the BigMAC exercise (two Permaflects panels and two Mystery panels) had recently been calibrated for accuracy. These panels were used to assess the accuracy of ASD- and UAS-based reflectance measurements. Different calibration procedures and traceability paths were used for the two sets of targets. This is described in detail in the following sections.

#### 3.2.1. Traceability

**Figure 10:**
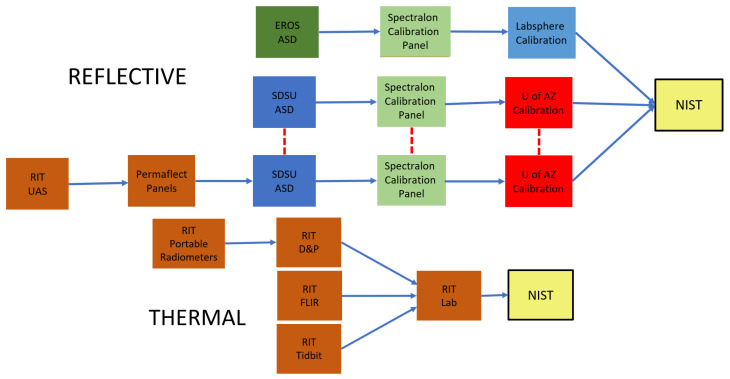
Traceability for reflective instruments (**top**) and thermal instruments (**bottom**).

Before looking at the accuracy data, the traceability of the instruments must be understood. Figure 10 shows the various instrument pathways to the NIST. The top half of Figure 10 shows the traceability pathway for the reflective instruments. Basically, there were two paths as follows: the USGS EROS ASD system was calibrated using a Labsphere spectralon calibration panel which was calibrated via the Labsphere calibration laboratory and adheres to the NIST Volunteer Laboratory Accreditation Program. The SDSU ASD system is traceable through its spectralon panel to calibration by the University of Arizona’s calibration laboratory. The RIT UAS reflective instruments were calibrated using the ELM based on observations of the Permaflect panels, as measured by the SDSU ASD. As a result, the traceability path is longer for the RIT UAS and has greater uncertainty attached to it.

Targets which had an absolute calibration attached to them included the Permaflect panels, which were calibrated at Labsphere, and the Mystery panels, which were calibrated at the University of Arizona. University of Arizona’s laboratory uncertainty varies from 0.0035 to 0.0065 absolute reflectance units depending on the wavelength. Labsphere’s uncertainty is similar for the Permaflect 55% panel at 0.0064 absolute reflectance units, on average, from the visible through the SWIR and up to 0.035 at 2200 nm. Because of the low signal level from the Permaflect 5% panel, traceability uncertainties were from 0.012 to 0.043 absolute reflectance units.

The absolute calibration placed on the Permaflect panels uses the diffuse reflectance method [34]. The absolute calibration placed on the Mystery panels included an illumination/viewing geometry that was almost identical to that which existed during the BigMAC 10:30 AM acquisitions. Because of this, the accuracy analysis was limited to only the morning acquisition.

#### 3.2.2. Approach

**Figure 11:**
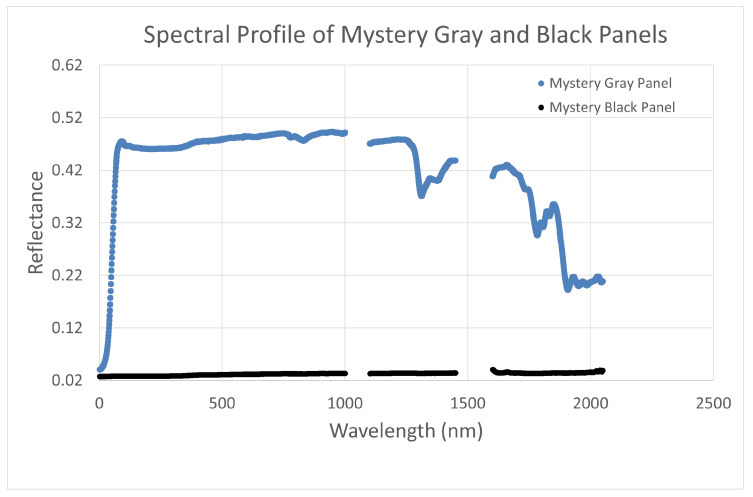
Spectral profile of Mystery panels.

The accuracy for the reflective measurement technologies was assessed using the Mystery panels and the Permaflect panels. Figure 9 and Figure 11 show the spectral profile of the Permaflect panels and Mystery panels, respectively. Calibration for the Mystery panels was available at 11 specific wavelengths. So, these were used to evaluate ASD and UAS measurements in a limited hyperspectral space. Calibration of the Permaflect panels was available from 350 to 2500 nm. So, for these targets, banded accuracy measurements were made to replicate the Landsat OLI spectral bands.

#### 3.2.3. Mystery Gray Panel Accuracy

**Figure 12:**
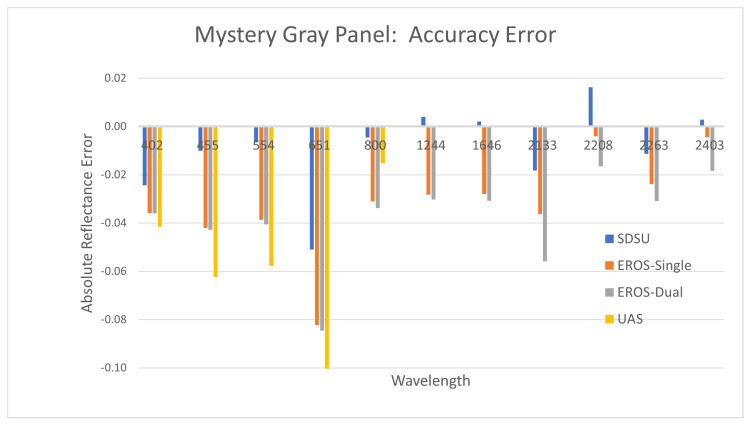
Reflectance measurement accuracy using the Mystery Gray panel.

Figure 12 provides the accuracy measurement results for the Mystery Gray panel, which had a reflectance from 0.22 to 0.50 dependent on wavelength. Each ASD instrument approach is included along with the UAS-based measurements. Unfortunately, the panel size was too small for the GSD of the UAS SWIR sensor, so data from these wavelengths are not included. The figure shows measured reflectance minus calibrated reflectance, or accuracy error. Results indicate typical accuracy for the SDSU ASD system of 0.02 reflectance units or less, while the EROS ASD systems were biased and slightly lower, at approximately 0.04 reflectance units. The UAS results were similar in bias to the EROS measurements but somewhat larger in magnitude.

#### 3.2.4. Mystery Black Panel Accuracy

**Figure 13:**
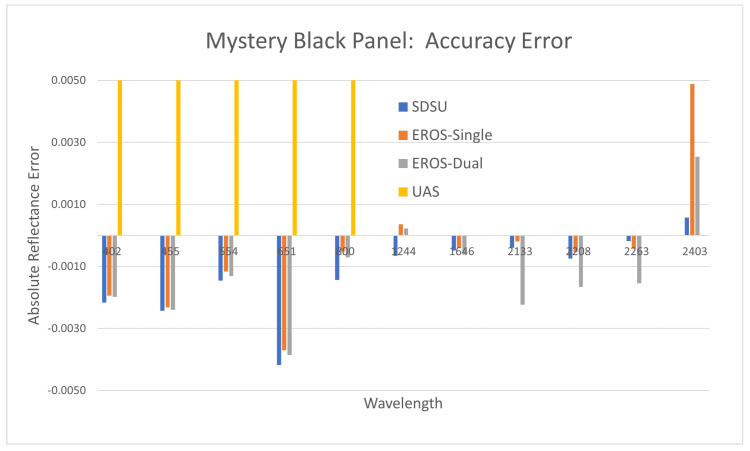
Reflectance measurement accuracy using the Mystery Black panel.

Figure 13 graphically shows the accuracy results for the Mystery Black panel, which had a calibrated reflectance between 0.030 and 0.035, dependent on wavelength. SWIR UAS measurements are again not available, as described previously. For this panel, ASD-based accuracy error was small, less than 0.005 reflectance units in all cases. However, UAS error was substantially larger (0.01 to 0.05 reflectance units) and biased in the opposite direction from the ASD-based measurements (the plot is scaled vertically to show the detail of the ASD measurement error). In general, ASD measurements are capable of achieving accuracies in the range of 0.002 reflectance units. These results also suggest that measurement accuracy is somewhat higher in the SWIR wavelengths compared to the VNIR wavelengths.

#### 3.2.5. 55% Permaflect Panel Accuracy

**Figure 14:**
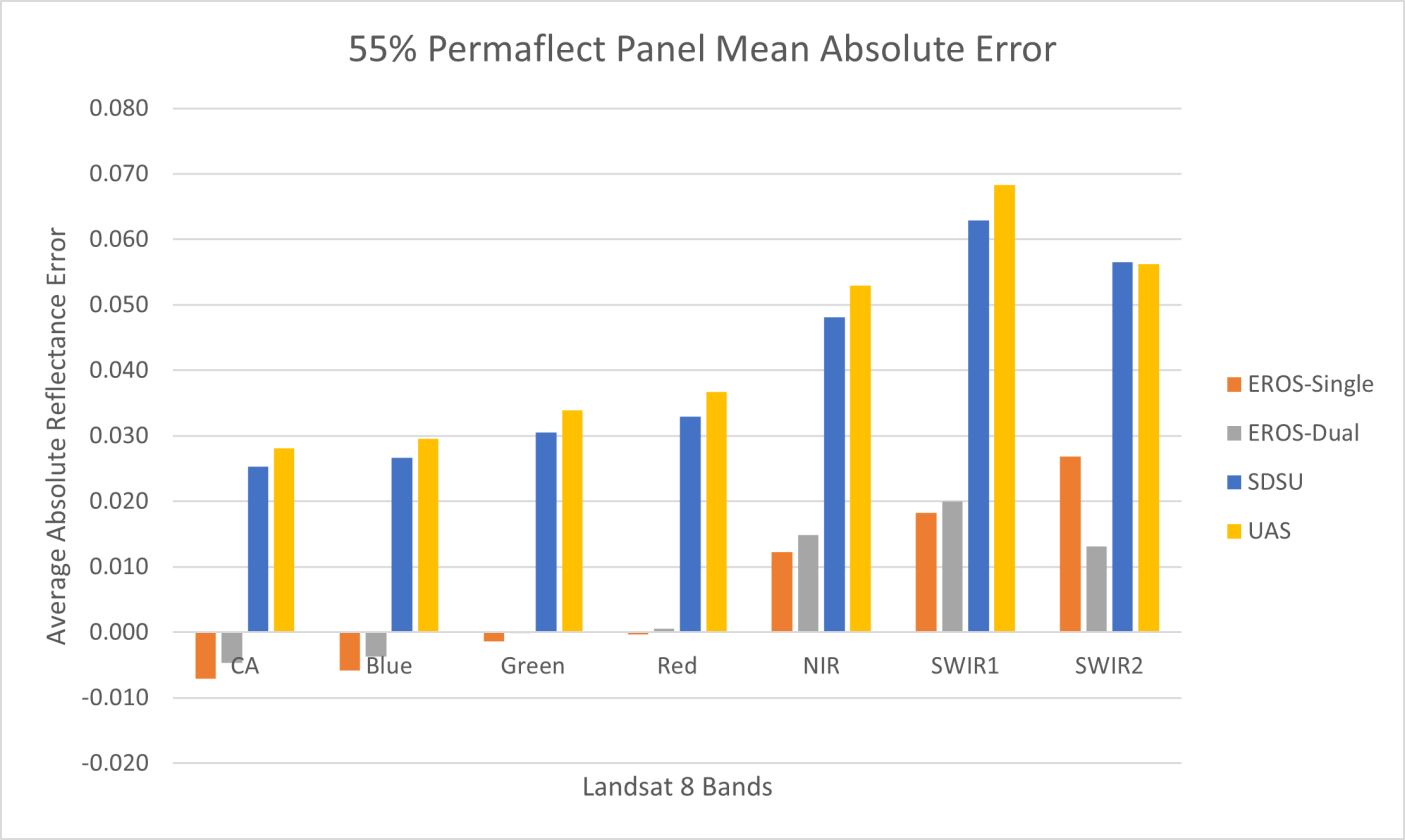
Reflectance measurement accuracy using the Permaflect 55% panel.

The accuracy error for the Permaflect 55% panel is shown in Figure 14. As the name suggests, the reflectance of this panel was close to 55% at all wavelengths. Accuracy errors tend to be small for the EROS ASD systems, with a positive trend as a function of wavelength; they range from −0.005 in the Coastal Aerosol band to +0.025 in the SWIR2 band. SDSU measurements are all biased positive and range from 0.025 to 0.065. UAS measurement errors are slightly larger than the SDSU errors. Since the UAS calibration was based on the ELM calibration approach using the SDSU measurement of the Permaflect panels, this result is consistent with the calibration traceability of the instrumentation. Overall, these results again indicate an accuracy of 0.02 reflectance units is possible with ASD-based systems.

#### 3.2.6. 5% Permaflect Panel Accuracy

**Figure 15:**
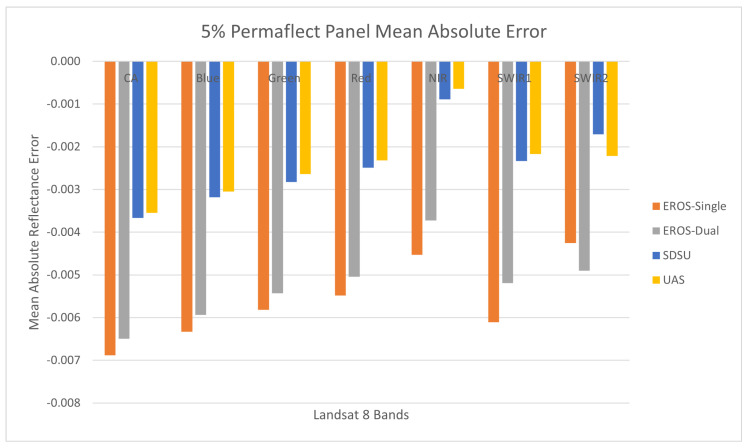
Reflectance measurement accuracy using the Permaflect 5% panel.

Figure 15 shows the accuracy error results for the 5% Permaflect panel. These results are unexpected in that they are all biased low, whereas the Permaflect 55% panel accuracy errors were all biased high. However, they are consistent in the sense that they shifted consistently on an absolute scale—the EROS results remain lower in value than the SDSU results. In this case, the EROS measurements have larger error than the SDSU measurements and range from approximately 0.003 to 0.007 while SDSU measurement errors are generally 0.003 or less. Surprisingly, the UAS results are the most accurate. However, these results are explained by the notion of accuracy and the traceability paths mentioned previously. In both cases, the Permaflect panels were calibrated using the same traceability path, and, relative to one another, the measurements have the same tendency—that is, SDSU measurements are larger than EROS measurements. For this particular panel, due to unknown causes, there is an overall bias present that has shifted the reference level.

#### 3.2.7. Accuracy Summary

To summarize the results for the accuracy assessment, the first point that needs to be made is that the traceability of the absolute calibration is a primary factor. Instruments that have been calibrated along a path consistent with the calibration of the targets under measurement tend to exhibit greater accuracy. For the case of the BigMAC campaign, this effect appears to be in the order of 0.02 reflectance units.

For the case of ASD-based measurements, absolute accuracy in the order of 0.02–0.03 reflectance units in the VSWIR seems achievable for bright targets (>0.30 reflectance units). Conversely, dark target results were somewhat mixed but tend to indicate accuracy in the order of 0.002 reflectance units. UAS accuracy measurements, as achieved for the BigMAC, were the lowest due to the traceability path inherent in the calibration of those sensors. With targets that are large with respect to the UAS instrument’s GSD, accuracy potential may be in a range from 0.02 to 0.04 reflectance units. For all instruments, it should also be noted that the reflectance of the surrounding area should also be similar to the target for the greatest accuracy. If the dissimilarity is too great, scattering from the surroundings will affect the accuracy measurements.

From an operational land surface reflectance product validation perspective, normally, targets will be large (relative to Landsat GSD), homogeneous areas. Thus, some of the difficulties present with small human-made reflectance panels will be mitigated. With the goal of a high degree of automation, it will likely be necessary to gather ground truth information on a regular basis to assess the stability of the deployed instruments. An example would be to send a team in the field with an ASD-based system and measure surface reflectance. Results from BigMAC indicate that accuracy using this approach, which has become essentially a de facto standard in the calibration community, will be approximately 0.02 reflectance units and could serve as an adequate baseline for the quality assessment of future land surface products.

## 4. Thermal Sensor Accuracy and Precision

### 4.1. UAS Sensors

Because of the difficulty involved in developing thermal targets that are stable over time, the results in this section will focus primarily on the accuracy and consistency across sensors during simultaneous measurements. Both UAS-borne and ground instruments will be compared. Primarily, due to the high degree of accuracy of the μFTIR and the TidbiTs, these will be used as reference sensors. Of particular interest will be the accuracy of the UAS FLIR sensor, as we aim to determine whether thermal imagery captured from a UAS platform can be used to validate higher-level products from satellite-borne systems.

### 4.2. Ground Sensors

Table 11 shows the key results that were generated from each flight. Recall that no flights occurred on the second afternoon due to high winds. Water targets are considered to be the most stable thermal target in the campaign and are highlighted in blue. These data suggest that the UAS FLIR is consistently within 1 °C of the TidbiT sensor in the pools. In comparison, the asphalt roadway temperature (highlighted in yellow) was measured by a contact thermocouple and the consistency with the UAS FLIR in this case was 2 °C. The thermocouples used in the experiment are base-metal Type K, with an accuracy of ±2.2 °C [35]. Lastly, the large alfalfa target results are highlighted in green. In this case, the UAS FLIR is within 1 °C of the ground-based measurement made by the μFTIR. Overall, the UAS FLIR instrument consistently provided 1 °C accuracy.

## 5. Mirror Empirical Line Method (MELM)

### 5.1. UAS Experiment

**Figure 16:**
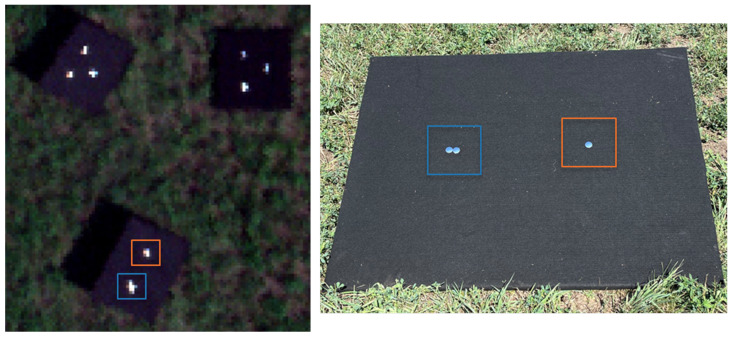
(**Left**) RGB composite image of the targets of interest. (**Right**) Ground photo of the targets. The colored boxes highlight the targets between the aerial and ground images.

During the primary BigMAC validation studies, a preliminary experiment on reflectance retrieval using SPARC technology was performed for UAS-based platforms. The goals of this experiment were to validate the perceived reflectance of the mirrors from calibrated imagery (i.e., from a diffuse reflectance-based ELM) and an initial application of a MELM on hyperspectral imagery. During the primary activities, the mirrors were deployed on black felt panels for isolating the mirror signal and optimal contrast; Figure 16. Because the mirrors are on a uniform background (felt panels), isolating the mirror signal is carried out by simply averaging the black felt signal.

The focus of this experiment was on the mirror signals highlighted in Figure 16, providing two points for the application of the MELM technique.

(1) $$\;LER\left(\lambda ,\theta_{i}^{m}\right)=\left[\frac{1}{\cos\theta_{o}}+\left(f-\frac{1}{\cos\theta_{o}}\right)G\left(\lambda \right)\right]\cdot \frac{N\pi R_{c}^{2}}{4\;GSD_{x}\;GSD_{y}}\cdot \rho_{m}\left(\lambda ,\theta_{i}^{m}\right)$$

where the following nomenclature holds: $\theta_{o}$Solar zenith angle (relative to a horizontal surface).$\theta_{i}^{m}$Solar angle of incidence on mirror surface (relative to mirror surface normal).$\rho_{m}\left(\lambda ,\theta_{i}^{m}\right)$Mirror specular reflectance spectrum measured in the laboratory at $\theta_{i}^{m}$.$R_{c}$Mirror radius of curvature.$GSD_{x}GSD_{y}$Along-scan and cross-scan ground sample distance of imaging sensor.*N*Number of identical mirrors contained in the SPARC target.$f=1-cos2\theta_{m}$Fraction of the hemispherical sky reflected by the mirror of
half angular width $\theta_{m}$ (measured from the center of curvature).G(λ)Diffuse-to-global irradiance ratio (on a horizontal surface) recorded
at the time of the image collect

**Figure 17:**
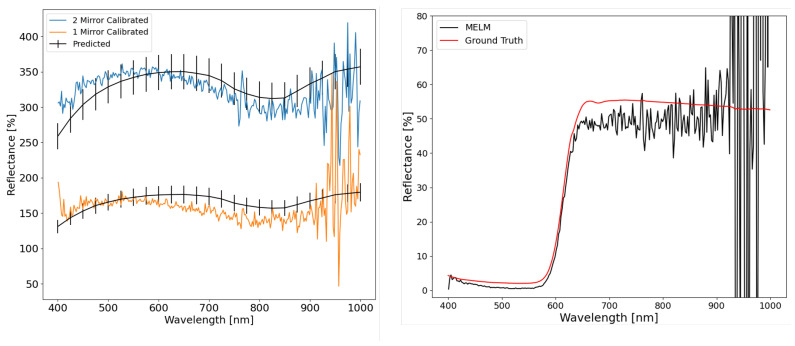
(**Left**) Validation of mirror reflectance with the colors corresponding to the mirrors in Figure 16. (**Right**) Two-point MELM applied to an in-scene red felt panel with ground truth data from a field spectrometer.

An initial validation of the mirror reflectance, or LER(λ), from the calibrated imagery was demonstrated to be within the expected uncertainty of 7.20% (Figure 17). This conservative uncertainty was calculated using the RSS method for the SPARC radiative transfer and LER(λ) equations [27]. The black curves are calculated using Equation (1) with N = 1 producing the predicted “low” LER SPARC target. Using N = 2 predicts the “high” LER SPARC target spectrum, both using the same specular reflectance spectrum, $\rho_{m}\left(\lambda ,\theta_{i}^{m}\right)$, of the mirrors measured in the laboratory. Both of the black curves with 7.20% error bars. For the validation, these are compared to the “high” (blue) and “low” (orange) spectra derived from the drone image data based on the gain and offset coefficients derived from the ELM analysis of the Permaflect Lambertian targets using the same two-point linear regression procedure in each spectral band.

The primary uncertainty contributions are the drone GSD, mirror reflectance factor, and focal length. The high LER(λ) of the mirror targets was expected but not designed for this UAS’s GSD. Due to a faulty lens iris, the f-number (f/♯) was larger than expected, which resulted in an overall low signal. Thus, the mirrors meant for a larger GSD were within the dynamic range of this sensor and equally valid for calibration. After validation, a two-point MELM technique was applied to the imagery and compared to the collected ground truth data for the RIT red felt target (Figure 17(right)). The difference in reflectance can be attributed to an inaccurate reflectance estimate of the black felt panel used as background for the mirrors. The background subtraction step is done by averaging the uniform dark background pixel radiance response around each target window (blue and red box in Figure 16) and subtracting that value from each pixel within the window to isolate the two-point calibration reference radiance form the reference targets. Thus, the background signal is simply an additive term to the scene reflectance that can be subtracted out when using the MELM technique [25] to isolate the calibration target signal in the image analysis. A strong BRDF component in the black felt can therefore account for the reflectance bias, though the spectral shapes were consistent. The BRDF bias can be the result because the drone image collection of the red felt panel (producing the MELM-derived spectrum shown in Figure 17, black curve on right) and the ASD spectroradiometer measured “ground truth” spectral reflectance factor collection were recorded at different times, so the illumination and view geometries were significantly different.

### 5.2. Sentinel-2A Experiment

**Figure 18:**
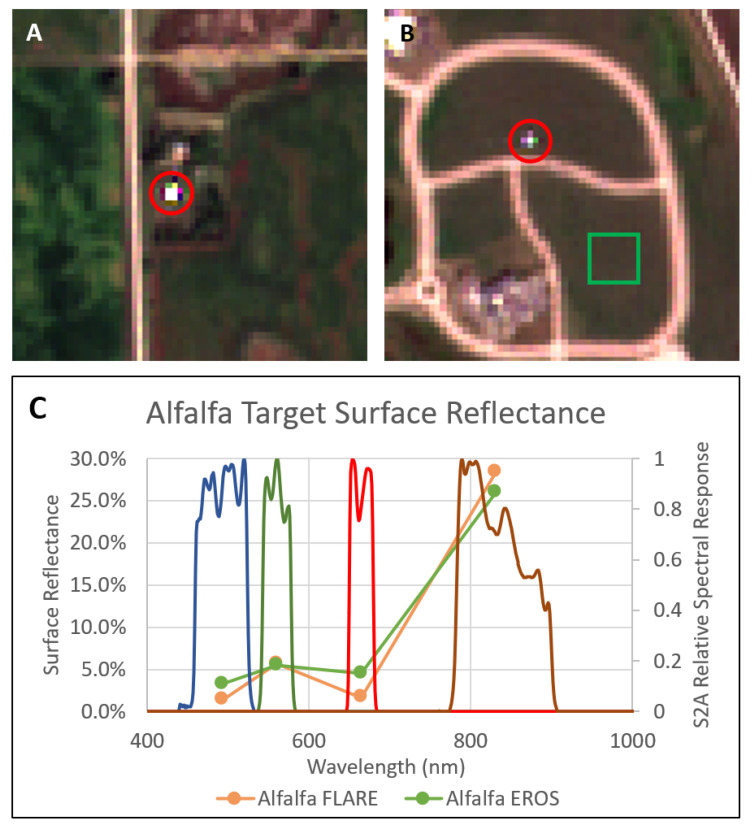
Automated FLARE station (**A**) and manual SPARC mirrors (**B**), indicated by the red circles, were utilized to perform a surface-reflectance MELM regression against satellite assets, particularly Sentinel-2A MSI, level 1C imagery. The derived gain coefficients were then applied to estimate the surface reflectance of the alfalfa field target at the BigMAC field site (green Region of Interest). The SPARC calibrated retrievals show reasonable agreement with in situ surface reflectance as measured by EROS (**C**). The blue, green, red, and brown curves represent the relative spectral responses of the Sentinel-2A MSI blue, greeen, red, and NIR bands, respectively.

**Figure 19:**
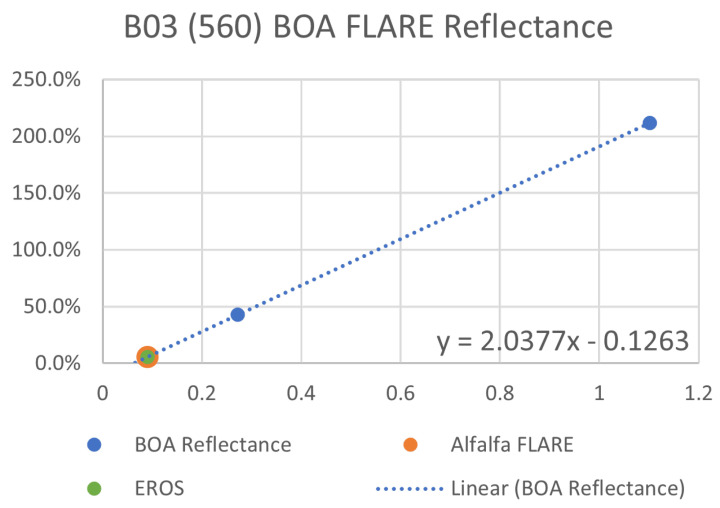
Example MELM surface reflectance regressions for Sentinel-2A MSI band 3 for a plot of at-sensor relative image radiance (x -axis) against calculated MELM target LER factor (y-axis). The SPARC target LER (blue dots) produce for each band a gain and offset that were used to derive the band-resolved spectral reflectance of the alfalfa test target pixels in Figure 18.

In addition to the primary BigMAC validation activities using the small point source and Lambertian reference panels with the UAS-based imaging systems, SPARC was used to target Sentinel-2A MSI 10 m bands during a coincident overpass (30 August 2021, 12:19 Local Time). The automated FLARE system (Figure 18A) and manual SPARC mirrors (Figure 18B) were used to generate high and low LER targets, respectively. Level 1C ToA reflectance imagery for the targets was downloaded (Copernicus Open Access Hub) and the mirror signals extracted using a proprietary software (Labsphere, Inc., North Sutton, NH, USA). These signals were then used to perform a MELM regression against the mirrors’ known LER (λ), producing the gain and offset factors used to convert the ToA imagery to a surface reflectance product (Figure 19) in the same manner as described for the UAS spectral data but convolved over the spectral response functions of the Sentinel-2A MSI bands (Figure 18C). Pixels from a region of interest corresponding to the BigMAC natural target area (Figure 18B, green square) were extracted, averaged, and compared to the ASD surface reflectance data measured during the experiment. When convolved against the Sentinel-2A MSI spectral response functions (Figure 18C), the ASD and Sentinel-2A surface reflectance values showed reasonable agreement over the VNIR range (Table 12). The agreement is within 6% except for band 08. The robust performance of the SPARC/FLARE system is illustrated by the fact that the two targets were separated over 30 km and it was able to extract the surface reflectance of the alfalfa field within expected uncertainties compared to ground truth measurements. This supports the capability for using the MELM method for producing reliable large area surface reflectance products.

## 6. Arable Radiometers

**Figure 20:**
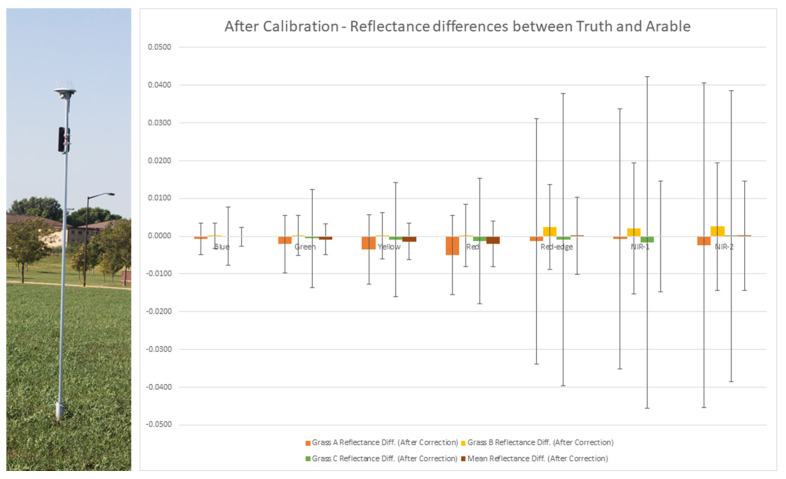
Photo of the deployed Arable radiometer on left. Plot of the Arable calibration accuracy on right. “Truth” refers to coincident spectroradiometer measurement.

Arable radiometers represent an economical approach for acquiring numerous automated surface reflectance measurements at many different locations. However, while their deployability and automation are highly attractive, their precision and accuracy remain a concern. The characterization of these devices over the past two years at SDSU provides some insights. Figure 20 shows a deployed Arable Mark 2 radiometer on a 10-foot pole and also a plot of calibration results. SDSU initial results indicate that the Arable Mark 2 is potentially capable of an accuracy with respect to ground truth measurements of 0.08 reflectance units if a single, uncalibrated unit is deployed over a growing season. If a minimum of three uncalibrated units are deployed, the accuracy improves through averaging to 0.04 reflectance units. Accuracy potential can be increased further if ground truth measurements are made over the field of view of the instrument at least two times during a growing season. Current analysis indicates that, while the absolute calibration may show an offset to absolute truth, the instruments appear to be stable over time but dependent on solar illumination angles. As such, the SDSU team is developing a methodology to evaluate angle dependency and calibrate the sensors using a series of measurements over the course of a one- to three-day period by taking measurements at various times/solar positions [32]. In this case, a single calibrated unit has been shown to be accurate to 0.005 reflectance units, and three calibrated units are accurate to 0.002 reflectance units. The plot in Figure 20 shows the accuracy for each spectral band based on these four levels of calibration.

## 7. Summary/Conclusions/Path Forward

### 7.1. Summary

With regard to precision, or repeatability, the results for the various technologies may be summarized as follows. The use of personnel carrying portable spectroradiometers has long been the standard for field measurements of surface reflectance. In the case of BigMAC, all teams used the ASD FieldSpec radiometer. For brighter targets, those in the range from 0.3 to 0.5 reflectance units, precision was in the range of 0.01–0.02 reflectance units. While, for darker targets, that is from 0.03 to 0.06 reflectance units, this approach was capable of measuring surface reflectance repeatably at 0.001–0.002 reflectance unit. For UAS-based measurements, the precision for brighter targets was comparable to the ground-based spectroradiometer results. However, for darker targets, the repeatability of UAS-based measurements increased to approximately 0.005. This difference may be due to, at least in part, the small physical size of the dark targets with respect to the UAS sensor’s GSD. Results derived from the larger, more pristine Permaflect panels were an indicator of the influence of target type on the measurements. This highlights the trade-off that is always present when using natural targets in the field versus highly developed human-made targets.

When considering the accuracy of surface reflectance measurements, the primary factor that must be understood is traceability. In the case of the BigMAC campaign, there were essentially two traceability paths. Differences in these paths led to at least a 0.02 reflectance unit difference among the sets of measurements defined by these two paths. For ground-based spectroradiometer measurements, accuracy in the range from 0.02 to 0.03 reflectance units was achievable for bright targets. Results for dark targets were somewhat mixed, but an accuracy of approximately 0.02 units was attainable. Because of the traceability path for the UAS-based radiometers, accuracy was lower than the ground-based radiometers. However, accuracy in the range from 0.02 to 0.04 reflectance units was attained.

Surface temperature measurements with an accuracy of 1–2 °K were attained in BigMAC. UAS-based measurements using the FLIR instrument recorded temperatures of water targets consistent with in situ TidbiT sensors, with an agreement of 1 °K. With respect to non-water targets, measurements of the FLIR instruments agreed with contact thermocouple instruments to 2 °K. These measurements are consistent with the specifications of the instruments reported previously. Additionally, measurements between the UAS-based FLIR sensor agreed with the μFTIR ground-based measurements to 1 °K.

Mirror-based measurements via the MELM are dependent on performing the background subtraction step accurately and on determining the equivalent diffuse reflectance of the mirrors. The MELM method results demonstrate consistency with the theoretical uncertainties of 7% when applied to UAS measurements. Results from other experiments indicate that this uncertainty can be greatly decreased with improvements in operation of the UAS sensor platforms. When using MELM for satellite observations in BigMAC, accuracies in the range of 0.02–0.03 reflectance units were attained, which is commensurate with differences observed for ground-based measurements between traceability paths. It should be noted that overcoming logistical constraints for satellite-based MELM during the BigMAC campaign could help yield improved results and this is the subject of ongoing work.

Lastly, the Arable Mark 2 radiometer can be used in conjunction with ground truth information to validate surface reflectance products. An accuracy of 0.002 reflectance units, with respect to the known ground truth, was demonstrated with arrays of at least three instruments at a site over the course of a single growing season.

### 7.2. Conclusions

BigMAC was conducted with the specific purpose of understanding how various surface reflectance and temperature measurement methodologies might be used to monitor surface reflectance/temperature in an operational manner to validate Landsat surface temperature and reflectance products. In particular, our study emphasized accuracy, precision, and deployability.

A large aspect of the campaign was the evaluation of handheld portable spectroradiometers that are normally deployed via a small team of people. Results from BigMAC are similar to those obtained in other campaigns [14,15]. From the perspective of accuracy and precision of measurement, this approach to surface reflectance has become a recognized standard in the community with regard to field campaigns. However, the deployability of this methodology remains expensive, requires multiple personnel, and can be quite time consuming.

UAS-based measurements of the surface reflectance were hampered by the target size in the BigMAC campaign, and due to the traceability of their calibration, the exhibited accuracy and precision predictably were slightly lower compared to the ground-based spectroradiometer method. Additionally, deployability typically involves several personnel and often has trailer-sized transportation requirements.

Thermal instrument accuracies of 1 K were obtained with the UAS/FLIR combination over land, as well as with the water-based TidbiT instruments. These are with respect to the μFTIR, with a laboratory-tested accuracy of 0.5%. Both of these methodologies are more than adequate with respect to accuracy for the validation of surface temperature products. However, deployability is substantially different: UAS deployment involves teams of people as well as support equipment, while TidbiTs can be left in a water environment for months unattended. However, additional data would be needed to fully understand the relations between water temperature measured by the TidbiT and surface temperatures of water recorded by a satellite-based instrument.

The MELM results show that this methodology can produce the repeatability and accuracy needed for surface reflectance product validation, as was demonstrated over a distance of several kilometers. However, further studies are needed to ensure that mirrors are properly designed and deployed and background subtraction is properly completed before the MELM is fully operational for validating surface reflectance products. Portable mirror systems are currently being designed and developed to address these issues.

The Arable Mark 2 radiometer can be used in conjunction with ground truth information to validate surface reflectance products. Two years of deployment by SDSU have demonstrated that the Arable Mark 2 radiometers can be fully automated in all but the most extreme environments; they are not able to withstand the extreme cold in South Dakota winters. However, they appear to be stable, characterizable instruments that can provide highly repeatable measurements with traceable accuracy over the course of a growing season with minimal maintenance through the visible and near infrared wavelength range.

### 7.3. Path Forward

The BigMAC results clearly indicated that the handheld portable spectroradiometer continues to provide highly accurate, repeatable measurements whenever ground truth is needed. However, it is not practical to use this technology over a widely distributed geographical region for a significant period of time due to the high time demands and costs associated with the field staff needed to complete these surveys. Thus, it remains a viable method to maintain the calibration of automated instruments deployed for long periods of time.

UAS deployed instrumentation is not feasible for similar reasons—high deployment costs. UAS deployments require a certified pilot, permits, and substantial investments in equipment and time to complete. UAS technology continues to improve and can potentially provide the prolonged cover of large areas. Therefore, reassessing deployments of UAS instrumentation in the future could help identify new ways to apply these methods.

TidbiTs can be used to collect simple, robust, and accurate water temperature data. However, additional study would be needed to optimize deployment for long term autonomous operation, as well as understand how the TidbiT measurement relates to the actual surface temperature of the water.

MELM can provide validation over many square kilometers of imagery but requires a portable mirror system that can work well with Landsat GSDs for operational deployment scenarios. Uncertainties have been demonstrated that meet the needs for surface reflectance validation but can be affected by background subtraction and the mirror design/size. Additional future studies could help optimize background subtraction and mirror design and size when using MELM. This is technology that needs to be monitored for future use.

Arable Mark 2 radiometers could be incorporated immediately to validate surface reflectance products. They could be widely deployed and would operate independently with little ground truth required over the course of a growing season, but they would have to be removed and stored in cold temperatures (sub 0 °C). Some effort will be required to ensure spectral differences can be addressed so they are compatible with Landsat spectral bands, and Arable Mark 2 radiometers are only limited to the VNIR wavelengths at this time.

BigMAC was not an exhaustive test of all available surface reflectance and temperature measurement technologies. Other methods are available and/or are being developed at the time of writing. The continued improvement of these technologies could result in more accurate surface reflectance and temperature products for the Earth remote sensing community.

## Figures and Tables

**Table 1:** Details of UAS flights conducted during BigMAC.

30 August 2021			
**Launch (UTC)**	**UAS Payload**	**Mode**	**Altitude (AGL)**
1539	MX-1	flightlines	60 m
1545	SWIR	flightlines	60 m
1615	MX-2	flightlines	60 m
1645	MX-2	stare	60 m
1716	MX-1	flightlines	60 m
1718	SWIR	flightlines	60 m
1735	MX-2	flightlines	60 m
1832	MX-2	stare	60 m
1913	MX-1	flightlines	60 m
1914	SWIR	flightlines	60 m
1916	MX-2	flightlines	60 m
2058	Mavic RGB	flightlines	60 m
**30 August 2021**	**Windy**		
**Launch (UTC)**	**UAS Payload**	**Mode**	**Altitude (AGL)**
1512	MX-1	flightlines	60 m
1513	SWIR	flightlines	60 m
1517	MX-2	flightlines	60 m
1704	MX-1	flightlines	60 m
1706	SWIR	flightlines	60 m

**Table 2:** Green band precision results for individual ASD systems.

**ASD Repeatability—Green Band**							
Bright Target Repeatability							
		Permaflect 55%	RIT 30%	Asphalt	Mystery Gray	Average	Std. Dev.
	Reflectance	0.51	0.30	0.13	0.49		
EROS Single	AM Difference	0.016	0.019	0.006	0.025	0.015	0.010
	Noon Difference	0.005	0.013	0.003	0.033		
EROS Dual	AM Difference	0.017	0.018	0.007	0.026	0.014	0.009
	Noon Difference	0.007	0.010	0.002	0.029		
SDSU	AM Difference	0.019	0.014	0.015	0.024	0.012	0.007
	Noon Difference	0.010	0.002	0.008	0.006		

Dark Target Repeatability							
		Permaflect 5%	RIT 2%	Alfalfa	Mystery Black	Average	Std. Dev.
	Reflectance	0.06	0.02	0.05	0.03		
EROS Single	AM Difference	0.001	0.002	0.002	0.000	0.001	0.001
	Noon Difference	0.000	0.001	0.002	0.001		
EROS Dual	AM Difference	0.001	0.002	0.002	0.000	0.001	0.001
	Noon Difference	0.001	0.001	0.002	0.001		
SDSU	AM Difference	0.001	0.001	0.002	0.000	0.002	0.001
	Noon Difference	0.001	0.001	0.002	0.003		

**Table 3:** Green band precision results across all ASD systems.

**ASD Repeatability Across All Teams—Green Band**									
Bright Target Repeatability									
		Permaflect 55%		RIT 30%		Asphalt		Mystery Gray	
	Reflectance	Mean	Std. Dev.	Mean	Std. Dev.	Mean	Std. Dev.	Mean	Std. Dev.
All Teams	AM	0.519	0.009	0.306	0.009	0.129	0.007	0.487	0.019
	Noon	0.512	0.011	0.303	0.007	0.126	0.004	0.498	0.019

Dark Target Repeatability									
		Permaflect 55%		RIT 30%		Asphalt		Mystery Gray	
	Reflectance	Mean	Std. Dev.	Mean	Std. Dev.	Mean	Std. Dev.	Mean	Std. Dev.
All Teams	AM	0.061	0.001	0.018	0.001	0.051	0.001	0.029	0.000
	Noon	0.058	0.001	0.018	0.001	0.055	0.002	0.028	0.001

**Table 4:** Green band precision results for UAS.

**UAS Repeatability—Green Band**						
Bright Target Repeatability						
	Permaflect 55%	RIT 30%	Asphalt	Mystery Gray	Average	Std. Dev.
Reflectance	0.52	0.31	0.14	0.46		
AM Difference	0.019	0.008	0.015	0.058	0.021	0.019
Noon Difference	0.010	0.006	0.004	0.046		

Dark Target Repeatability						
	Permaflect 5%	RIT 2%	Alfalfa	Mystery Black	Average	Std. Dev.
Reflectance	0.06	0.02	0.06	0.04		
AM Difference	0.001	0.003	0.008	0.017	0.005	0.005
Noon Difference	0.001	0.001	0.008	0.003		

**Table 5:** NIR band precision results for individual ASD systems.

**ASD Repeatability—NIR Band**							
Bright Target Repeatability							
		Permaflect 55%	RIT 30%	RIT Red	Mystery Gray	Average	Std. Dev.
	Reflectance	0.53	0.38	0.53	0.51		
EROS Single	AM Difference	0.015	0.029	0.035	0.023	0.021	0.008
	Noon Difference	0.007	0.018	0.020	0.026		
EROS Dual	AM Difference	0.015	0.029	0.035	0.023	0.020	0.008
	Noon Difference	0.009	0.013	0.012	0.023		
SDSU	AM Difference	0.021	0.016	0.033	0.019	0.014	0.010
	Noon Difference	0.012	0.003	0.002	0.005		

Dark Target Repeatability							
		Permaflect 5%	RIT 2%		Mystery Black	Average	Std. Dev.
	Reflectance	0.06	0.02		0.03		
EROS Single	AM Difference	0.000	0.002		0.002	0.001	0.001
	Noon Difference	0.000	0.001		0.003		
EROS Dual	AM Difference	0.001	0.002		0.001	0.001	0.001
	Noon Difference	0.000	0.001		0.003		
SDSU	AM Difference	0.001	0.002		0.000	0.001	0.001
	Noon Difference	0.000	0.001		0.003		

**Table 6:** NIR band precision results across all ASD systems.

**ASD Repeatability Across All Teams—NIR Band**									
Bright Target Repeatability									
		Permaflect 55%		RIT 30%		RIT Red		Mystery Gray	
	Reflectance	Mean	Std. Dev.	Mean	Std. Dev.	Mean	Std. Dev.	Mean	Std. Dev.
All Teams	AM	0.547	0.009	0.390	0.013	0.542	0.019	0.502	0.017
	Noon	0.535	0.011	0.388	0.009	0.534	0.013	0.506	0.016

Dark Target Repeatability									
		Permaflect 5%		RIT 2%				Mystery Black	
	Reflectance	Mean	Std. Dev.	Mean	Std. Dev.			Mean	Std. Dev.
All Teams	AM	0.057	0.001	0.020	0.001			0.030	0.001
	Noon	0.054	0.001	0.019	0.001			0.030	0.002

**Table 7:** NIR band precision results for UAS.

**UAS Consistency—NIR Band**						
**Bright Target Repeatability**						
	Permaflect 55%	RIT 30%	RIT Red	Mystery Gray	Average	Std. Dev.
Reflectance	0.55	0.40	0.55	0.51		
AM Difference	0.021	0.006	0.043	0.120	0.035	0.036
Noon Difference	0.012	0.004	0.017	0.056		
					Std. Dev. w/o mystery gray:	0.013

**Dark Target Repeatability**						
	Permaflect 5%	RIT 2%		Mystery Black	Average	Std. Dev.
Reflectance	0.06	0.06		0.08		
AM Difference	0.001	0.011		0.059	0.016	0.021
Noon Difference	0.000	0.004		0.020		
					Std. Dev. w/o mystery black:	0.005

**Table 8:** SWIR2 band precision results for individual ASD systems.

**ASD Repeatability—SWIR2 Band**							
Bright Target Repeatability							
		Permaflect 55%	RIT 30%	RIT Red	Mystery Gray	Average	Std. Dev.
	Reflectance	0.43	0.37	0.39	0.32		
EROS Single	AM Difference	0.018	0.035	0.027	0.037	0.023	0.010
	Noon Difference	0.007	0.022	0.009	0.028		
EROS Dual	AM Difference	0.016	0.034	0.026	0.035	0.020	0.011
	Noon Difference	0.005	0.017	0.004	0.026		
SDSU	AM Difference	0.023	0.023	0.036	0.004	0.016	0.010
	Noon Difference	0.005	0.007	0.014	0.013		

Dark Target Repeatability							
		Permaflect 5%	RIT 2%		Mystery Black	Average	Std. Dev.
	Reflectance	0.05	0.03		0.03		
EROS Single	AM Difference	0.000	0.003		0.002	0.001	0.001
	Noon Difference	0.000	0.002		0.002		
EROS Dual	AM Difference	0.000	0.003		0.002	0.001	0.001
	Noon Difference	0.000	0.002		0.001		
SDSU	AM Difference	0.002	0.003		0.001	0.002	0.001
	Noon Difference	0.001	0.001		0.005		

**Table 9:** SWIR2 band precision results across all ASD systems.

**ASD Repeatability Across All Teams—SWIR2 Band**									
Bright Target Repeatability									
		Permaflect 55%		RIT 30%		RIT Red		Mystery Gray	
	Reflectance	Mean	Std. Dev.	Mean	Std. Dev.	Mean	Std. Dev.	Mean	Std. Dev.
All Teams	AM	0.440	0.012	0.371	0.017	0.402	0.017	0.321	0.019
	Noon	0.436	0.012	0.375	0.013	0.387	0.013	0.318	0.016

Dark Target Repeatability									
		Permaflect 5%		RIT 2%				Mystery Black	
	Reflectance	Mean	Std. Dev.	Mean	Std. Dev.			Mean	Std. Dev.
All Teams	AM	0.051	0.001	0.028	0.002			0.034	0.001
	Noon	0.049	0.001	0.028	0.001			0.034	0.002

**Table 10:** SWIR2 band precision results for UAS.

**UAS Repeatability–SWIR2 Band**						
Bright Target Repeatability						
	Permaflect 55%	RIT 30%	RIT Red		Average	Std. Dev.
Reflectance	0.438	0.354	0.389			
AM Difference	0.000	0.093 *	0.083		0.062	0.044
Noon Difference	0.005	0.073	0.120			

Dark Target Repeatability						
	Permaflect 5%	RIT 2%			Average	Std. Dev.
Reflectance	0.050	0.036				
AM Difference	0.004	0.025 *				
Noon Difference	0.001	0.028 *				

Note: * indicates inconsistent measurements.

**Table 11:** Thermal sensor accuracy measurements.

Flight 1—Morning—30 August 2021 (Estimated Pass 11:23 )			
Object	Time (CDT)	Ground-reference temperature (C)	FLIR temperature (C)
Concrete (contact thermocouple)	11:23 AM	36.13	33.94
ColdPool (TidBit)	11:23 AM	13.83	14.12
AmbientPool (TidBit)	11:23 AM	25.76	24.92
**Flight 2—Morning—Stare—30 August 2021 (Estimated Pass 11:52)**			
Object	Time (CDT)	Ground-reference temperature (C) [Std. Dev]	FLIR temperature (C) [Std. Dev]
Concrete (contact thermocouple)	11:52 AM	38.83	37.39
ColdPool (TidBit)	11:52 AM	15.14 [0.125]	15.28 [0.202]
AmbientPool (TidBit)	11:52 AM	26.3 [0.062]	25.62 [0.495]
**Flight 3—Noon—30 August 2021 (Estimated Pass 12:33)**			
Object	Time (CDT)	Ground-reference temperature (C)	FLIR temperature (C)
Concrete (contact thermocouple)	12:33 PM	42.30	40.49
ColdPool (TidBit)	12:33 PM	13.43	13.11
AmbientPool (TidBit)	12:33 PM	27.07	25.12
**Flight 4—Afternoon—30 August 2021—Stare—(Estimated Pass 13.37)**			
Object	Time (CDT)	Ground-reference temperature (C) [Std. Dev]	FLIR temperature (C) [Std. Dev]
Concrete (contact thermocouple)	1:37 PM	45.08	43.00
ColdPool (TidBit)	1:37 PM	11.07 [0.028]	12.51 [0.268]
AmbientPool (TidBit)	1:37 PM	27.9 [0.046]	24.16 [0.366]
Alfalfa (μFTIR)	1:35 PM	37.91 [1.63]	38.62 [2.05]
**Flight 5—Afternoon—30 August 2021 (Estimated Pass 14:21)**			
Object	Time (CDT)	Ground-reference temperature (C)	FLIR temperature (C)
Concrete (contact thermocouple)	14:21 PM	45.83	43.62
ColdPool (TidBit)	14:21 PM	13.01	14.19
AmbientPool (TidBit)	14:21 PM	28.38	24.66

**Table 12:** Alfalfa surface reflectance derived from the SPARC calibrated Sentinel-2A surface reflectance product and in situ measurements resolved to Sentinel-2A bands.

Surface Reflectance			
Sentinel-2A MSI Band	CW (nm)	FLARE Sentinel-2A Image	EROS Ground Truth
02	493	1.4%	3.3%
03	560	5.7%	5.5%
04	665	1.6%	4.5%
08	831	28.4%	26.0%

## Data Availability

The data presented in this study, collected by USGS, are openly available in ScienceBase at https://doi.org/10.5066/P9E3F6LV, accessed on 10 December 2024.

## Abbreviations

The following abbreviations are used in this manuscript:

AGL	Above Ground Level
ASD	Analytical Spectral Device
ASOS	Automated Surface Observing System
BigMAC	Big Multi-Agency Campaign
BRDF	Bidirectional Reflectance Distribution Function
ELM	Empirical Line Method
EROS	Earth Resources Observation and Science
FLARE	Field Line of sight Automated Radiance Exposure Network
FLIR	Forward Looking Infrared
FTIR	Fourier Transform Infrared
GSD	Ground Sampling Distance
LaSRC	Land Surface Reflectance Code
LER	Lambertian Equivalent Reflectance
MELM	Mirror-based Empirical Line Method
MSI	Multi-Spectral Instrument
NIR	Near Infrared
NIST	National Institute for Standards and Testing
OLI	Operational Land Imager
RAIFoV	Radiometrically Accurate Instantaneous Field of View
RCR	Remote Cosine Receptor
RIT	Rochester Institute of Technology
RoC	Radius of Curvature
RSS	Root Sum Square
SDSU	South Dakota State University
SI	International System of Units
SPARC	SPecular Array Radiometric Calibration
SWIR	Shortwave Infrared
ToA	Top of Atmosphere
UAS	Unmanned Aircraft System
USGS	U.S. Geological Survey
VNIR	Visible Near Infrared
VSWIR	Visible Shortwave Infrared
GPS	Global Positioning System
IMU	Inertial Measurement Unit
FOR	Field-of-Regard
